# Predictive Modelling for Concrete Failure at Anchorages Using Machine Learning Techniques

**DOI:** 10.3390/ma14010062

**Published:** 2020-12-25

**Authors:** Panagiotis Spyridis, Oladimeji B. Olalusi

**Affiliations:** 1 Faculty of Architecture and Civil Engineering, Technical University of Dortmund, 44227 Dortmund, Germany; 2 Structural Engineering & Computational Mechanics Group (SECM), Department of Civil Engineering, University of KwaZulu-Natal, Durban 4001, South Africa; olalusio@ukzn.ac.za

**Keywords:** concrete, fracture, anchorage, fasteners, machine learning, support vector machine, Gaussian process regression

## Abstract

Anchorage to concrete plays a significant role in various aspects of modern construction. The structural performance of anchors under direct tensile load can lead to failure by concrete cone breakout. Concrete related failure modes are quasi-brittle, and as such, they may develop without prior warning indications of damage, while it also exposes the bearing component to damage propagation. As such, an adequate reliability assessment of anchors against concrete cone failure is of high importance, and improved precision and minimisation of uncertainty in the predictive model are critical. This contribution develops predictive models for the tensile breakout capacity of fastening systems in concrete using the Gaussian Process Regression (GPR) and the Support Vector Regression (SVR) machine learning (ML) algorithms. The models were developed utilising a set of 864 experimental anchor tests. The efficiency of the developed models is assessed by statistical comparison to the state-of-practice semi-empirical predictive model, which is embedded in international design standards. Furthermore, the algorithms were evaluated based on a newly introduced Model Explainability concept based on Analogous Rational and Mechanical phenomena (MEARM). Finally, a discussion is provided regarding the developed ML models’ suitability for use as General Probabilistic Models in a reliability framework.

## 1. Introduction

Anchorage to concrete plays a vital role in various aspects of modern construction. Many applications are structural connections, such as foundations of steel columns to concrete blocks or rafts, steel girders on concrete cores of high-rise buildings, assemblies of precast elements, and a multitude of reinforced interfaces and integrations of strengthening components to existing structures [1]. In many cases, anchorages are used for the stability of temporary works, for example, the fixation of falsework and scaffolding. Other safety-critical applications include non-structural fixtures, for example, in residential, healthcare, civil protection, or industrial buildings, such as facades, suspended ceilings, utilities, electrical devices and engines. Also, in linear infrastructure projects, a multitude of fixings is widely used to support noise barriers, rails, cabling, to name a few. Relevant design guidelines have been published to underpin a rational design, specification, and usage of fastenings, with the new Eurocode 2—Part 4 (EN 1992-4) [2] being the most notable recent standardisation initiative. The American counterpart, the ACI-318, has already been including design provisions for anchorage to concrete for many years, and these provisions were transferred from a code appendix to an individual chapter incorporated with the code [3] in 2013. While the American and European standards serve as a basis for further national regulations, supplementary standardisation documents for fastenings to concrete are also introduced at the national level worldwide [4,5,6].

Depending on the currently applicable design criteria, the load resistance of anchors is determined by semi-empirical formulations for each potential failure mode, based on the type of load (e.g., static or dynamic) and orientation (axial or shear) as well as other structural and geometric parameters. The design is then typically based on a semi-probabilistic (partial safety factor) design concept. Each calculation is carried out for the resistance against a specific failure mode, considering a factored load, a factored equation, and the involved characteristic material parameters (e.g., steel, concrete, adhesives). The purpose is to provide adequate safety reserves and to compensate for the respective variabilities, within which also a model uncertainty underlies [1,2,3,7].

In quasi-static situations, failure modes for single anchors in tension may be steel rupture, concrete cone breakout, pull-out (or combined cone breakout and pull-out, e.g., for bonded and concrete screw anchors), splitting, and side blow-out, or failure of the rebar within the concrete component. From a life-cycle perspective, additional causes of failure, such as corrosion or fatigue, can interfere, but also overloading or underperformance of the concrete substrate can occur. Concrete related failure modes are of particular interest for two main reasons: (a) they are quasi-brittle, and as such, they may develop without preceding signs of damage, so there is limited possibility for remediation action and avoidance of complete system failure; (b) concrete failure leads to extensive damage in the area of the fastening, and there is limited possibility for adequate repair [1]. This consequently leads to a particular interest in the adequate assessment of such systems’ reliability in the case of concrete cone failure. This study refers to single anchor experiements with geometrical attributes leading exclusively to concrete related failure modes. In particular, this is concrete cone breakout under tension, while there is also reference to concrete edge failure under shear toward the edge.

Although industry digitalisation and artificial intelligence led to a boom in the application of advanced soft computing systems in all sectors in recent years, the field has been evolving since the 1950s. “Knowledge-based” approach or “expert systems” have been used to reduce the effects of indetermination in various scientific and industrial disciplines. Applications within the structural engineering field have been seen since the 1980s [8,9,10]. Since then, ML techniques have often been used to model structural engineering problems. They also extend to assessments in the structural reliability realm, such as downscaling the computing effort of stochastic simulations [11,12] and performance evaluating of structural systems of high complexity [9,13,14]. Some of these studies have delivered interesting results on the application of ML and other soft computing techniques to model steel-concrete connection engineering problems. Golafshani et al. [15] discuss ANN models in comparison with Fuzzy Logic (FL) for the prediction of the bond strength of spliced rebar. The dataset of 179 tests considered was randomly split to 125, 27 and 27 data points for training, validating, and testing purposes, respectively. Both the ANN and FL models predicted the bond strength with high accuracy (*R*^2^ > 0.99 in all cases).

Sakla and Ashour [16] elaborated 1143 quasi-static tensile tests on single-bonded anchors as a subset of an international database reported in [17]. The authors predicted the ultimate load using a single hidden layer ANN model. The training set comprised 88.3% of the entire dataset and achieved a prediction precision of the test set by 1.03 and a coefficient of determination *R*^2^ = 0.941. Ashour and Alqedra [18] investigated the breakout resistance of single anchors utilising a dataset of 451 quasi-static tests of single anchors in uncracked concrete. Of the entire dataset, 225 data points were used for the training of four different ANNs, with five to seven hidden layers each. The predictions by use of the ANNs captured the test results with mean ratios between 1.025 to 1.065, standard deviations between 19.9% and 22.6% and coefficients of determination *R*^2^ varying between 0.879 and 0.907, and they indicated that the Concrete Capacity Design (CCD) method is a reliable predictor of the ultimate loads. An investigation by the same authors [19] on the shear capacity of single anchors failing under edge breakout has led to a similar conclusion comparing the CCD method [20] to predictions from ANN. A total of 205 experimental tests were considered in this investigation. The mean value of the ratio of the estimated to the actual strength is 1.054 with a standard deviation of 22% and a coefficient of determination *R*^2^ = 0.886, while the predictive model of [20] only achieves an *R*^2^ = 0.768.

Gesoğlu and Güneyisi [21] performed a re-evaluation of the database from [17] using 932 training and 177 testing data from tension tests on adhesive anchors. The analysis used an ANN algorithm in order to propose an improved calibration of the CCD equations used in the codified design. For anchors failing with a concrete cone failure, the mean value of the calculated to measured values for the testing dataset are 0.99 and 1.63 for the models based on ANN and the CCD method, respectively. A complementary study in [22] including an analysis by ANN on the same dataset, indicated that an ANN-based model delivered still a higher accuracy with correlation coefficients of 0.983 and 0.984 with the training and testing data, respectively. The fact that the correlation with both the testing and training data is virtually the same, allowed to conclude that the ANN has an overall more reliable prediction performance. The correlation coefficient of the tests to predictions based on the models used in [2,3] was 0.885 and 0.883, respectively. Some of the key issues facing the efficient implementation of ANN include the difficulty in obtaining the optimum hyperparameters, slow convergence speed, over-fitting problem, difficulty in obtaining suitable network topology and poor generalisation capability [23,24]. While the ANN algorithm has been implemented in a variety of previous studies involving the concrete breakout strength of anchors in tension, more powerful and reliable ML algorithms should be studied for a more extensive and up to date experimental database.

Recent investigations present soft computing techniques such as the Gaussian Process Regression (GPR) [25,26], and the Support Vector Regression (SVR) [10,27,28] with very high efficiency. This is discussed by [29], which concluded that both modelling techniques deliver superior predictive accuracy than ANNs and semi-empirical models proposed in current design standards, which are based on nonlinear statistical regression (NR) [30,31,32]. Besides SVR and GPR, other ML techniques including ensemble models (including random forests), Adaptive neuro-fuzzy inference systems, Bayesian networks, have been studied as possible candidates for efficient predictive models.

This contribution aims to propose ML-based strength models for accurate prediction of the concrete breakout strength of single anchors loaded in tension. Among the algorithms investigated, the GPR and SVR are shown to optimally capture the resistance values. Firstly, the structural behaviour of anchors failing due to the concrete cone is briefly discussed. A description of the GPR and SVR algorithms and the background of the assessment conducted are discussed in Section 2.2. Using an extensive and up-to-date database of 864 tests on single anchors failing due to concrete cone breakout, developed by the American Concrete Institute Scientific Committee 355—Anchorage to concrete, the models are trained and tested, and the best-ranking candidate models are qualified (Section 3). A comparative evaluation of the developed models against experimentally observed tensile breakout strength is performed and discussed based on a novel ranking methodology. An additional criterion for the model qualification is Model Explainability based on Analogous Rational and Mechanical phenomena (MEARM), which is for the first time reported, trialled, and applied herein on structural engineering problems. The qualified ML models are evaluated in terms of precision and performance relative to the state-of-the-art predictive model embedded in international design codes (Section 4), and their suitability for use as General Probabilistic Models is discussed, which introduces the possibility for alternative, more accurate design methodologies in a reliability framework.

### 1.1. Behaviour of Anchors in Tension Subject to Concrete Cone Failure

The structural performance of anchors under direct tensile load can lead to concrete cone failure, which is the main interest of this study. One of the seminal approaches and the one currently applied in most design standards is the so-called Capacity Design (CCD) Method [20]. This design method was established based on the fracture mechanical theory and a substantial set of tensile experiments on anchors with a range of material and geometrical parameters. The anchor types that can be primarily considered for this failure mode are mechanical post-installed or cast-in headed anchors. Still, it is understood that under the circumstances, this failure can also occur for bonded or expansion anchors, or other types of fastening products. This type of failure typically governs the load-bearing performance of an anchorage for certain combinations of concrete and steel strengths, along with certain geometrical arrangements, such as anchor embedment depth, concrete member thickness, and edge distance. The resistance is also affected by the existence of cracks and reinforcement in concrete (see also [1] for an in-depth explanation of the various influences). The failure is assumed to initiate at the lower end of the anchor and propagate to the surface at an angle of 30° to 40° to the horizontal. On average, the angle is 35°, and thus an idealised breakout area can be assumed with a size equal to 3·$h_{ef}$ (the embedment depth).

According to the mechanical concept of the CCD Method, the concrete cone breakout failure load can be calculated using the various forms of Equation (1).

(1) $$N={\left(k_{1}\cdot f_{c}\right)}^{0.5}\cdot {\left(k_{2}\cdot h_{ef}\right)}^{2}\cdot {\left(k_{3}\cdot h_{ef}\right)}^{-0.5}$$

where, N is the tensile breakout resistance; $k_{1},\;k_{2},\;k_{3}$ are calibration factors; $h_{ef}$ is the anchor embedment depth (the distance of the failure invitation point to the free surface); $f_{c}$ is the concrete compressive strength.

**Figure 1:**
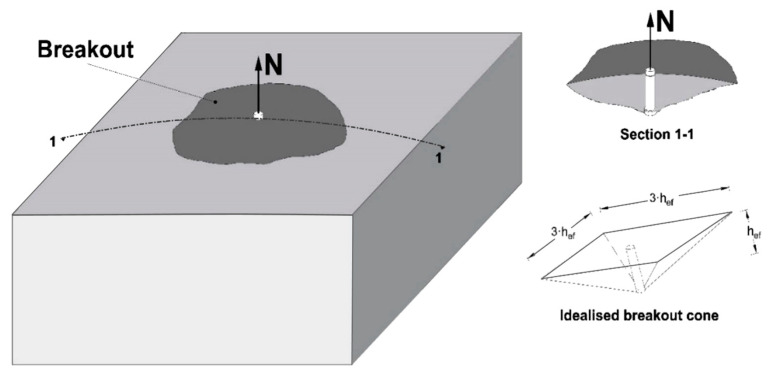
Sketch of concrete cone failure, and dimensions of the idealised cone.

In Equation (1), the factor $k_{1}\cdot f_{c}{}^{0.5}$ represents the tensile capacity of concrete. The factor $k_{2}\cdot h_{ef}$ represents the geometry of the failure surface. The factor $k_{3}\cdot h_{ef}$ signifies the size effect on concrete tensile failure. According to the analytical approach in [33,34], the concrete tensile strength is the main defining factor for the failure surface generator and the breakout capacity. To estimate the mean capacity of a single anchor according to [35], in plain uncracked concrete without edge influence, Equation (2) can be used. Studies presented in [1], indicated that the influence of a crack with a width beyond 0.4 mm leads to a reduction in the anchor resistance by 30% to 50% (average 40%). For undercut anchors and headed studs, this seems to stabilise for large crack widths, failing with concrete breakout. This formula also accounts for a tensile stress regime in the anchor vicinity, transverse to its axis. For design purposes, EN1992-4 proposes the calculation of the characteristic resistance through Equation (3). The product-specific value $k_{ucr}$ can be replaced by $k_{cr}$ to estimate the strength of an anchor in cracked concrete, assuming a crack width of 0.2 mm. The recommended values are $k_{cr}=7.7$ and $k_{ucr}=11.0$ for post-installed anchors, while $k_{cr}=8.9$ and $k_{ucr}=12.7$ for cast-in headed studs [7]. The projected area of the breakout on the free surface of the concrete body is used as an adjustment indicator in the case of a group or an anchor with influence from the concrete boundaries, as explained in detail in [1]. The idealised unaffected concrete breakout body is also depicted in Figure 1.

(2) $$N_{Rm,c}^{0}=k_{c}\cdot h_{ef}{}^{1.5}\cdot \sqrt{\alpha_{c}\cdot f_{cc}}\;[N]$$

where, $N_{Rm,c}^{0}$ is the mean tensile breakout resistance; $k_{c}$ is equal to the value of 13.5 for metal expansion anchors and bonded anchors; $\alpha_{c}$ is equal to the value of 1.0 for an undisturbed uniaxial compression stress state (this occurs for $h_{ef}$ equal to approximately 5 anchor diameters) is equal to the value of 0.8 for anchors within compression zones with cracks developing parallel to the compression direction (this occurs for $h_{ef}$ greater than 5 anchor diameters); $f_{cc}$ is the mean compressive strength measured at 200 mm concrete cube specimens.

(3) $$N_{Rk,c}^{0}=k_{ucr}\cdot h_{ef}{}^{1.5}\cdot \sqrt{f_{ck,cube}}\;[N]$$

where, $N_{Rk,c}^{0}$ is the characteristic tensile breakout resistance; $k_{ucr}$ is a product-specific value for non-cracked concrete; $f_{ck,cube}$ is the characteristic compressive strength from 150 mm concrete cube specimens.

### 1.2. Analogies to Shear Concrete Edge Failure

**Figure 2:**
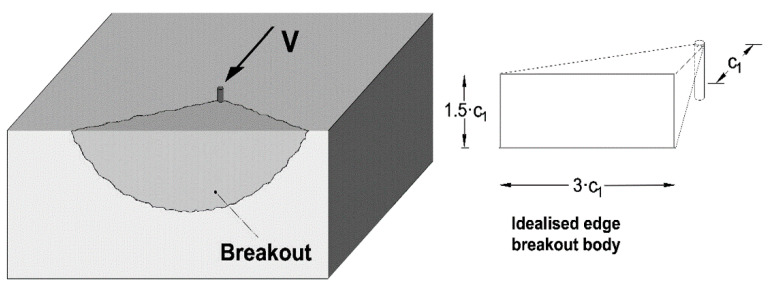
Sketch of concrete edge breakout for an anchor under shear with a load toward the edge and dimensions of idealised failure body.

Concrete edge failure under a transverse load toward the edge presents a similarity to the concrete cone failure. Concrete edge failure (or breakout) occurs particularly for anchorages close to a free edge of the concrete component, and it develops from a fracture initiating at the upper part of the anchor propagating to detachment of a half-cone shaped concrete prism mobilised by the transversely loaded anchor (Figure 2). The shear load capacity of single anchors can be evaluated from Equation (4), which is the updated CCD formulation presented in [32]. Comparing Equation (1) assuming cracked concrete and (4), the same fractural and size-effect mechanistic principles are evident when the embedment depth $h_{ef}$ (height of a cone split in half by a crack plane) is replaced by the distance from the concrete edge $c_{1}$ (height of the half-cone). The theoretical equivalence between the mechanical models of concrete breakout under shear and axial load is also mentioned in [20], and it is evident in the dimensions of the failure prisms indicated in Figure 1 and Figure 2. In Equation (4), it is also apparent that the anchor diameter and stiffness have some influence on the shear failure load. This influence is estimated to be in the range of 20%, based on a recalculation of the anchor configurations in an extensive experimental database discussed in [29].

(4) $$V_{Rm,c}^{0}=3\times d_{nom}^{\alpha }.l_{f}^{\beta }.\sqrt{f_{cm}.}c_{1}^{1.5}\;[N]$$

where

$$\alpha =0.1\times {\left(\frac{l_{f}}{c_{1}}\right)}^{0.5}$$

$$\beta =0.1\times {\left(\frac{d_{nom}}{c_{1}}\right)}^{0.2}$$

$d_{nom}$ is the outside diameter of the anchor; $f_{cm}$ is the mean concrete cylinder compressive strength; $l_{f}$ is the influence length of the anchor loaded in shear; $c_{1}$ is the concrete edge distance; $V_{Rm,c}^{0}$ is the mean shear breakout resistance.

In [29], the applicability using GPR and SVR techniques to predict the concrete cone breakout capacity of single anchors loaded in shear is investigated. The predictive efficiency of the algorithms is also compared with that of results from various nonlinear regression models currently proposed as design equations, as well as results from ANN algorithms by other researchers. It is concluded that both SVR and GPR can deliver a more precise prediction compared to formulations currently used in design standards. Furthermore, it is made evident that the GPR model is the best predictor of all the models assessed, over the entire dataset, but also over anchor configurations with extreme geometrical characteristics, i.e., those with the minimum and maximum anchor diameter, embedment depth, and distance from the edge. These ML techniques, among others, are also discussed below for their applicability to anchors under tensile loading, leading to concrete cone failure.

## 2. Development of the ML-Based Predictive Models

### 2.1. Processing and Preparation of the Experimental Database

**Figure 3:**
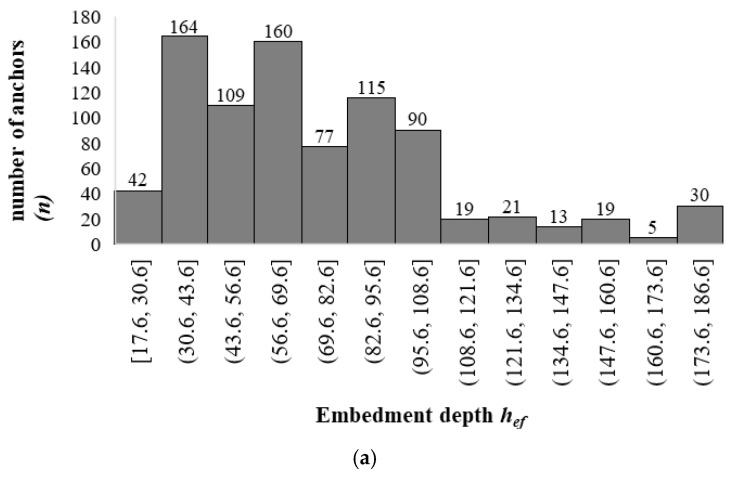

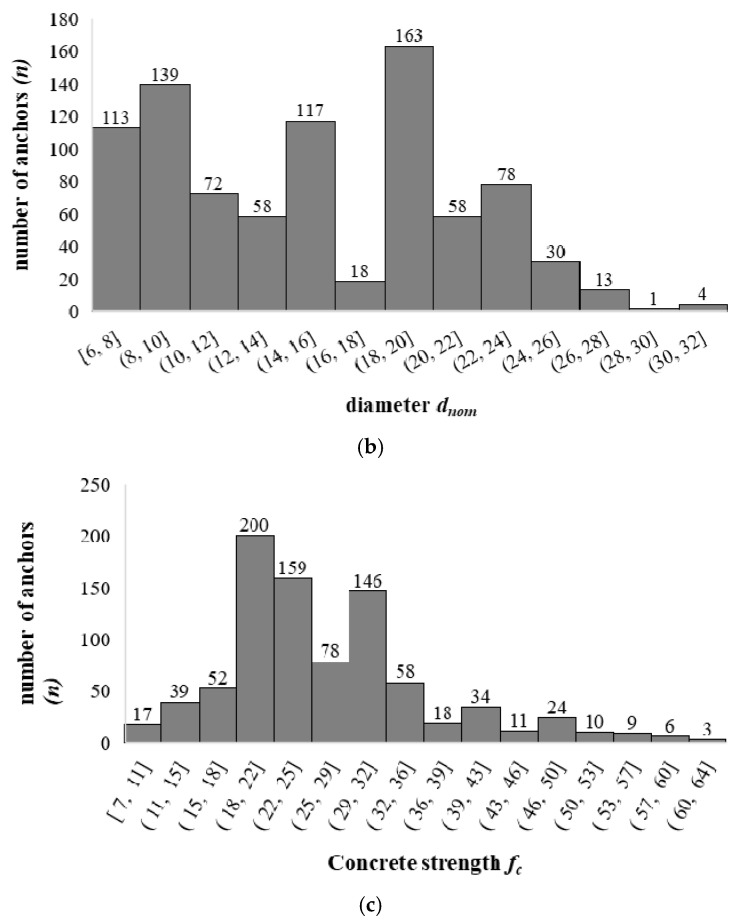
Histograms of the data representation. (**a**) Embedment depth; (**b**) Anchor diameter; (**c**) Concrete strength.

The database considered in this investigation was compiled by the American Concrete Institute Scientific Committee 355—Anchorage to concrete. It contains the observed experimental failure load from 864 concrete breakout tests for different types of tensile loaded single anchors in uncracked concrete. At the same time, the data used are tests failing with unrestrained concrete breakout failure. A summary of the database is presented in Table 1, while the distribution and histograms of the design parameters are illustrated in Figure 3.

As shown in Figure 3a, the majority of the anchor tests in the database, 88% (757 tests), had an embedment depth of $h_{ef}\leq 110\;\mathrm{mm}$, however, 12% of the anchor tests have embedment depth $h_{ef}$ between 110 and 190 mm (Figure 3a). Figure 3b revealed that most anchor tests 80% (691 tests) have concrete strength $f_{c}\leq 32\;\mathrm{MPa}$. A total of 816 anchors (94%) featured diameters of $d_{nom}$ ≤ 24 mm. There are only a small number of experimental tests on larger diameter anchors with $d_{nom}$ > 24 mm (6% (48 anchors)) (Figure 3c). Generally, the dataset is deemed representative of the majority of real applications, and of design situations covered by current design standards.

The most widely accepted methods (i.e., the various versions of the CCD method) suggest that the critical variables influencing the concrete breakout strength of tensile loaded anchors are the embedment depth $h_{ef}$, the anchor diameter $d_{nom}$ and the concrete strength $f_{c}$. These parameters are considered as the input variables for the proposed ML-based models developed in this study. Some further influences are discussed in the literature, such as the boundary and stress state of the concrete specimen [36,37], the aggregate size, the aggregate and cement constituents [37,38,39], and the concrete age [38]. However, these influences are reported as minor or negligible, captured already by the use of compressive strength as a predictor, while very few test data are available. Therefore, these influences were not accounted for in the input dataset.

To implement the ML algorithms, the database was divided into two subsets: data set training and testing. The training dataset is applied to develop the models, whereas the testing dataset is utilised to evaluate the efficacy of the developed models. When dividing the database into subsets, it is important to maintain statistically consistent data patterns for both the training and testing datasets. This was accomplished by randomly splitting the database until there was an appropriate consistency between the input parameters of the testing and training dataset in terms of statistical properties presented in Table 2. In this study, 75% of the data (648 out of 864 tests) were adopted for training, and the remainder (216 tests) were used for testing the developed models.

### 2.2. Machine Learning (ML) Techniques

#### 2.2.1. Gaussian Process Regression (GPR)

The Gaussian process algorithm presents a probabilistic, nonparametric supervised learning technique for classification and regression problems. This ML technique received a great deal of attention due to its ability and efficiency in handling nonlinear data using kernel functions. Previous studies [25,26,40,41,42,43] have shown that the Gaussian process regression GPR can provide efficient and reliable predictions. GP has various forms of possible kernel functions (similar to the SVM), which includes rational quadratic kernel, exponential kernel, squared exponential kernel/RBF and Matern 5/2. This study employs the RBF kernel for developing a concrete cone breakout strength prediction model for anchors in tension, which was found to provide the best data fit, out of several trialled kernel functions. A detailed overview of the GPR methodology can be found in [42,44].

#### 2.2.2. Support Vector Regression (SVR)

The support vector machine (SVR) was developed by Vapnik and Chervonenkis in 1964. The machine learning technique is recognised as an appropriate tool to solve classification and regression related problems. The support vector machine is a nonparametric, supervised learning approach for regression and classification problems. Highly efficient and stable performance of SVR for regression related problems has been demonstrated in [10,27,28,45]. Compared to other machine learning algorithms, the SVR method adopts the structural risk minimisation (SRM) principle, which simultaneously minimises the error on the training set and maximises the generalisation ability of the model. Other machine learning algorithms such as ANN employs an empirical risk minimisation (ERM) principle, which only minimises error on the training set and is more prone to over-fitting [46,47]. The common kernel functions used in SVR include the gaussian or radial basis function (RBF), sigmoid, polynomial and linear. Based on fitting trials of several kernel functions, the polynomial kernel function was used for developing the prediction model reported herein.

#### 2.2.3. Other Algorithms Considered

A series of other algorithms have been considered as possible predictive models. These included ensemble models Random Forests, Decision Trees and Neural Networks. Examination of these algorithms in terms of fitting to the training data and predicting the test data has indicated that they are not efficient or of further benefit to the study. The outcome of the examination is briefly discussed in Section 3, but they are not further described herein. A detailed discussion of these types of algorithms can be found in [48,49].

### 2.3. Implementation of the ML Algorithms

In this study, the Gaussian process and the support vector regression algorithms are implemented using a MATLAB environment. The model development task involved training and testing of the dataset. Each test case in the training dataset contains one “target value” and several “input values”. The three input variables considered for developing the models are concrete strength, anchor diameter and embedment depth. The single output variable was taken as the experimentally observed concrete cone breakout strength of a single tensile loaded anchor. The Z-score normalisation technique was adopted to normalise the data so as to prevent variables in higher numeric ranges from dominating those in smaller numeric ranges [29,43].

There are four common kernels functions for GPR and SVR. Taking into consideration that specific kernel functions are suitable for a given dataset type, the different kernel functions were evaluated in order to identify the most suitable kernel functions. Based on the evaluation, the two most suitable kernel functions obtained for the database are the Radial Basis Function (RBF) and polynomial kernel function for the GPR and SVR and model, respectively. The two kernel functions have less numerical difficulties compared to the other kernel functions. The implementation of ML algorithms includes the selection of the right set of algorithm design parameters. The identification of the optimal/best values of the design parameters was made using the optimum search method in MATLAB. A 5-fold cross-validation technique is adopted.

### 2.4. Performance Evaluation Measures

The efficiency of the proposed models is investigated in terms of different statistical parameters, namely the mean and coefficient of variation of the ratio of the predicted to experimentally observed concrete breakout strength (model uncertainty), coefficient of determination (*R*^2^) (Equation (5)), mean absolute error (*MAE*) (Equation (6)) and root-mean-squared error (*RMSE*) (Equation (7)). The model quality increases when the mean ratio of the predicted results to the experimentally observed results and *R*^2^ value approaches one, while the values of *MAE*, CoV and *RMSE* decrease (approach zero).

(5) $$R^{2}=1-\frac{\sum_{i=1}^{n}{\left(N_{pred}-N_{exp}\right)}^{2}\;}{\sum_{i=1}^{n}{\left(N_{pred}\right)}^{2}}$$

(6) $$MAE=\left(\frac{\sum_{i=1}^{n}\left|N_{pred}-N_{exp}\right|}{n}\right)$$

(7) $$RMSE=\sqrt{\frac{1}{n}\overset{n}{\underset{i=1}{\sum }}{\left(N_{pred}-N_{experiment}\right)}^{2}}$$

where $N_{exp}$ and $N_{pred}$ are values of measured and predicted values and n is the number of data samples.

## 3. Results and Discussion

### 3.1. Performance Evaluation of the Developed Models: Comparison of GPR and SVR Model Predictions to Experimental Data

In this study, various machine learning algorithms are trialled, and the outcome is summarised in Table 3. From the table, it was clear that the GPR and SVR models achieved the best results in terms of the highest determination coefficient for both the training and testing dataset. They also have the lowest mean square error amongst the five functions. Hence, the GPR and SVR algorithms were adopted for predicting the tensile breakout resistance. The detailed assessment of the accuracy of the proposed GPR and SVR models using the experimental dataset is discussed in this section.

**Figure 4:**
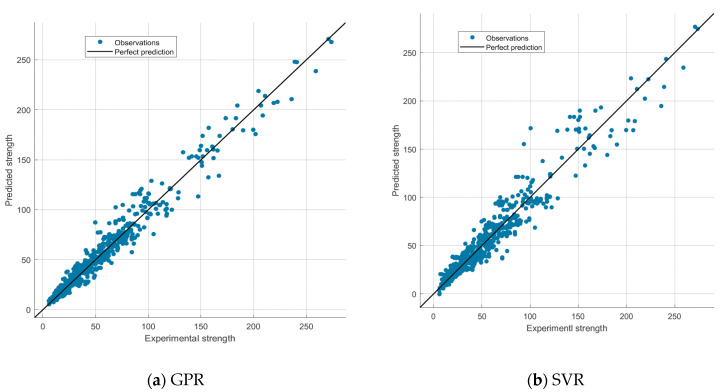
Plot of predicted tensile strength (kN) against experimentally observed strength (kN) using the training datasets. (**a**) GPR; (**b**) SVR.

**Figure 5:**
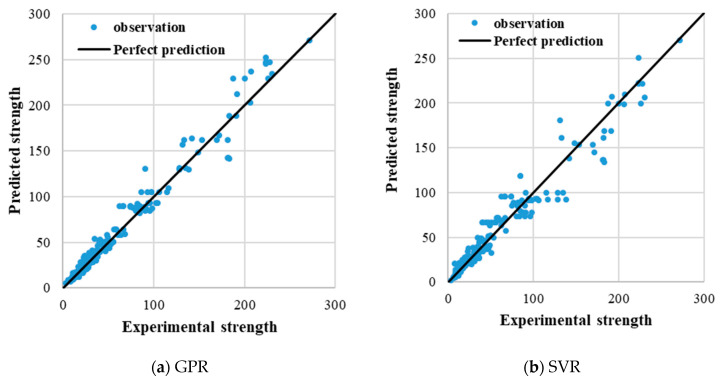
Plot of predicted strength (kN) against experimentally observed strength (kN) using the testing datasets. (**a**) GPR; (**b**) SVR.

**Figure 6:**
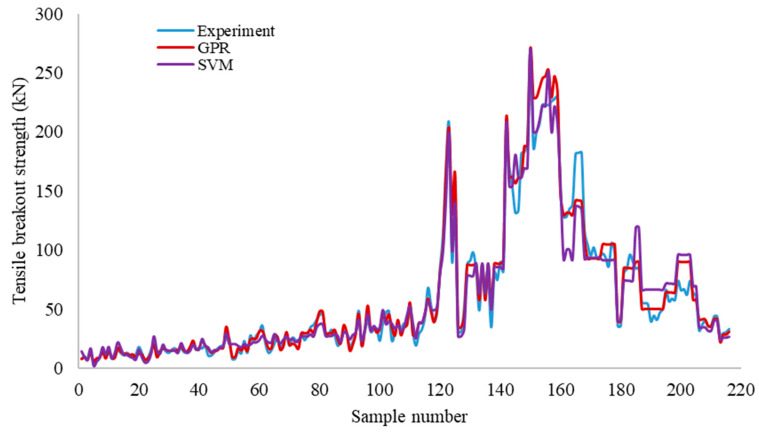
Comparison of predictions from the ML-based models to the experimental values using the testing dataset.

The plots of the predicted versus the experimental breakout strength for the training dataset and testing dataset are presented in Figure 4 and Figure 5, respectively, in order to demonstrate the performance of the developed GPR and SVR models. Figure 6 further elucidates the comparison between the experimentally obtained and the predicted concrete breakout strength. The predictions of the proposed models are well correlated with the experimental breakout strength, as illustrated in the figures.

The statistical metrics *R*^2^, RMSE and MAE were obtained using Equations (5)–(7) and the values summarised in Table 3. As seen in the table, the estimated *R*^2^, RMSE and MAE values between the observed values and predicted values of the GPR model are 0.97, 8.15 and 5.56, respectively, for the testing dataset. The estimated *R*^2^, RMSE and MAE values for the SVR predictive model are 0.93, 11.73 and 7.98, respectively, for the testing dataset. The values of the statistical metrics estimated for both the testing and training database are comparable. Generally, the results presented in this study demonstrate that both the GPR and SVR models captured the experimental data reasonably well. In particular, the GPR model yielded the best result in both the training and testing dataset.

### 3.2. Influence of Input Variables on Model Performance

Since the GPR and the SVR models yielded the best performance, the influence of their basic input variables on their performance was assessed and discussed in this section. To do this, each input variable in the database is omitted one at a time, and the resulting dataset is used to train and test the ML algorithms. The performances of the proposed models using the resultant database are presented in Table 3. The table revealed that all the input variables are influencing the predicted tensile breakout strength of single anchors in concrete. However, compared to other input variables the concrete strength $f_{c}$ and embedment depth $h_{ef}$ are the most sensitive variables affecting the breakout strength of tensile loaded anchors for both models. It can be noted that omitting the anchor diameter $d_{nom}$ results in a minimal change in the performance of the developed models. The result is consistent with the CCD approach, which ignores the influence of the anchor diameter, as discussed in Section 1.1 and presented in [20].

### 3.3. Comparison of the Developed ML Based Predictions to Existing Methods

**Figure 7:**
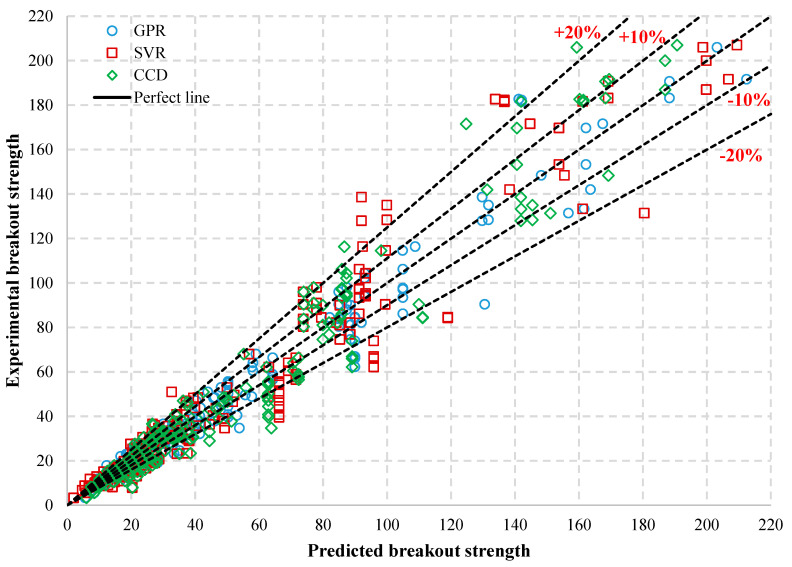
Predicted value versus the experimental value of concrete breakout capacities for the developed models and Concrete Capacity Design (CCD) method.

The CCD method, proposed by Fuchs et al. [20], is the most used and widely accepted basic method for predicting the tensile concrete cone breakout strength of single anchors in concrete. The CCD method is currently incorporated into several international design standards such as ACI 318-08 [3], CEN/TS 1992-4 [2], indicating that the method is well accepted. Predictions from the GPR and SVR models are compared to that of the CCD method to evaluate the efficacy of the developed models using the testing database. The predictions from the developed ML-based models are compared with the prediction accuracy of the CCD method in terms of the mean and coefficient of variation of the ratio of the predicted to experimentally observed concrete breakout strength (model uncertainty), $R^{2}$, RMSE and MAE. The plot of the experimental concrete breakout resistance against the predicted resistance by GPR, SVR and the CCD model, using the testing database (Table 2), are presented in Figure 7. It may be observed from the figure that the GPR predictions are less scattered compared to the other approaches and more within ±10% of the equality/perfect line. The predictions by the SVR and CCD model are broadly dispersed, especially above the ±10% line of equality.

In Table 4, the RMSE and MAE values of the GPR model are the lowest of all the models investigated. The GPR model has an *R*^2^ value of 0.97, which is the closest to the value of one, out of all the models considered. Given the results presented in Table 4 and Figure 7, the GPR model reasonably predicted the experimental data with higher accuracy and more consistently than the other models considered in this study.

**Figure 8:**
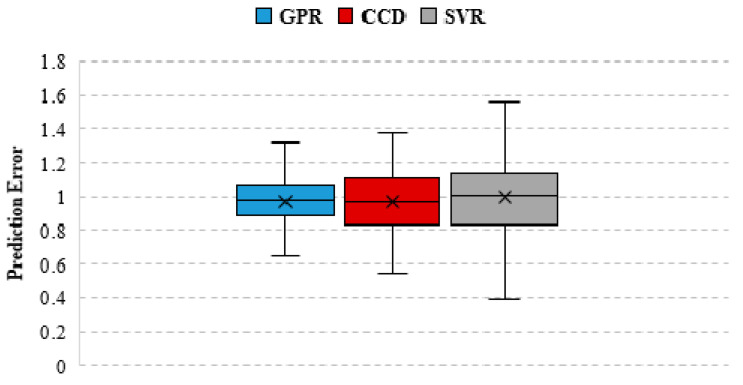
Comparison of the prediction error statistics for the developed machine learning models (GPR, SVR) and the existing semi-empirical predictive equation (CCD).

A statistical comparison is also obtained based on the ratios of the experimental tensile breakout resistance, to the one predicted by the models, and the results are included in Table 4. The respective distributions are graphically presented in a box plot shown in Figure 8. The ratios are considered in this instance since they can be translated into more suitable expressions of model uncertainties [48,49,50]. The model uncertainty variable associated with the GPR model has a mean value of $\mu_{M}=0.97$. The predictions from the GPR model produced the lowest dispersion with $\sigma_{ME}=0.14$. The SVR model has the largest dispersion with $\sigma_{M}=0.22$, but the experimental to predicted ratios obtained for the SVR model have a mean value of $\mu_{M}=0.99$, which is closest to the optimal value of 1, as compared to the other models. Considering also the statistical parameters *R*^2^, RMSE, and MAE, it is evident that, although the accuracy of the SVR model is higher than the other models, the overall precision of the predictions from the GPR model exceeds the one of the SVR as well as the CCD model.

To facilitate evaluation and selection of the most suitable predictive model, a rationalised model ranking method, proposed in [50], is applied to the investigated models, which uses the experimental to predicted ratio parameters shown in Table 4. With this method, models are ranked, based on (i) $\mu_{M}$ closest to 1.0, (ii) least $\sigma_{M}$ and (iii) minimum ratio of $(M_{max}$/$M_{min})$. The overall ranking is the average of rankings (i), (ii), and (iii).

Based on this system, the GPR model ranks first, followed by the SVR model and the CCD model. As seen in Table 4, although this ranking uses the statistical data of the ratios, it is also in agreement with the statistical indicators based on the residuals, RMSE and *R*^2^.

Ashour and Alqedra [18] proposed an ANN-based model for concrete cone breakout resistance of single anchors in tension using a database of 451 tests. The performance of the model was evaluated for different networks using the statistics of the model uncertainty (mean value $\mu_{M}$ and dispersion $\sigma_{M}$). The authors obtained a mean value in the range of $\mu_{ME}=1.03–1.07$ and dispersion in the range of $\sigma_{ME}=0.20–0.23$ for the various networks of ANN considered. A similar investigation was conducted by Gesoğlu and Güneyisi [21]. The analysis was conducted using the ANN algorithm and Genetic Programming technique. For anchors failing with a concrete cone failure, the mean of the model uncertainty variables for the testing dataset is 0.99 for the models based on ANN. The obtained standard deviation is 0.16 for the ANN-based model. Evidently, the proposed GPR model predictions outperform the ANN model reported by [18,21]. However, this must remain under the perspective that [18] has used a smaller dataset and that [21] has used a more complex dataset and fastening system associating with more than one failure mode (adhesive anchors).

## 4. Model Explainability Based on Analogous Rational and Mechanical Phenomena (MEARM)

The comparison of the results of the developed models to the already tested and widely acknowledged practical model of the CCD method indicates that the ML models have a trend in predicting the test results with similar statistical performance. This is explained in more depth in the following section. Although the qualified ML models are shown to perform better predictions, the statistical moments of the errors are in the same order of magnitude (Table 4). The respective distributions are also generally bell-shaped, uniform, with a single maximum and with small skewness. This is an indication that the ML models also agree with the CCD method, which is in turn confirmed by mechanical fracture engineering theories (See also Section 1.1 and Section 2.1), and as such exhibit an explainable description of the concrete cone failure resistance.

A significant finding is furthermore that the same algorithms qualified herein, GPR and SVR, are the ones that have shown the best performance in predicting the concrete edge failures under shear in the investigations by the same authors in [29]. For the SVR algorithm, even the best fitting kernel function (polynomial) coincides for both the tension and shear datasets. Also, the deviations from the experiment predictions had again similar statistical characteristics. The modes of failure under edge and cone breakout under shear and tension pose fundamental mechanistic similarities, which are discussed in Section 1.2. Nevertheless, the testing conditions and configurations, as well as the resistance values differ significantly, while [29] showed that shear resistance is associated with a higher number of predictors than with tension. Therefore, the algorithmic coincidence is not anticipated in principle. The phenomenological and algorithmic coincidence in the analyses by use of the ML indicates that the structure of the commonly fitting GPR and SVR algorithms can also describe the mechanical phenomenon described by the algorithms. This accordance is defined herein as Model Explainability based on Analogous Rational and Mechanical phenomena (MEARM). It is proposed as an additional rational criterion for the selection of applicable ML models, and it reinforces the selection of the GPR and SVR algorithms as predictive models for the resistance of anchors against concrete breakout failure modes in particular.

## 5. Implementation of the Machine Learning Algorithms as General Probabilistic Models

### 5.1. Association of Predictive Efficiency to Model Uncertainty Characteristics

Model uncertainty can be understood as a description of the model’s deficiency in accurately describing a physical phenomenon (in the present case, the anchor’s resistance) due to lack of knowledge in the problem description, or to mathematical simplifications. In the realm of structural reliability, it is expressed as a random variable accounting for the abovementioned effects, and it is expected to have a major influence in reliability analysis [51,52]. As such, it can be directly linked to the statistical characteristics of the prediction errors discussed in the previous section, with model uncertainty ($\theta_{x})$ determined by Equation (8) [50,51,52]. Apparently, model uncertainty $\theta_{x}=1$ is a condition for an ideal model, $\theta_{x}>1$ implies that the model underpredicts, and $\theta_{x}<1$ that it overpredicts the actual concrete breakout resistance.

(8) θx=Nexp,x Npred, x(X)

where, $N_{exp,x}$ is the experimentally observed failure load for a single anchor test x in tension; X represents the input variable set, which includes the embedment depth $h_{ef}$, anchor $d_{nom}$ and the concrete strength $f_{c}$; $N_{pred,\;x}$ represents mean concrete breakout model prediction for the same anchor test x.

**Figure 9:**
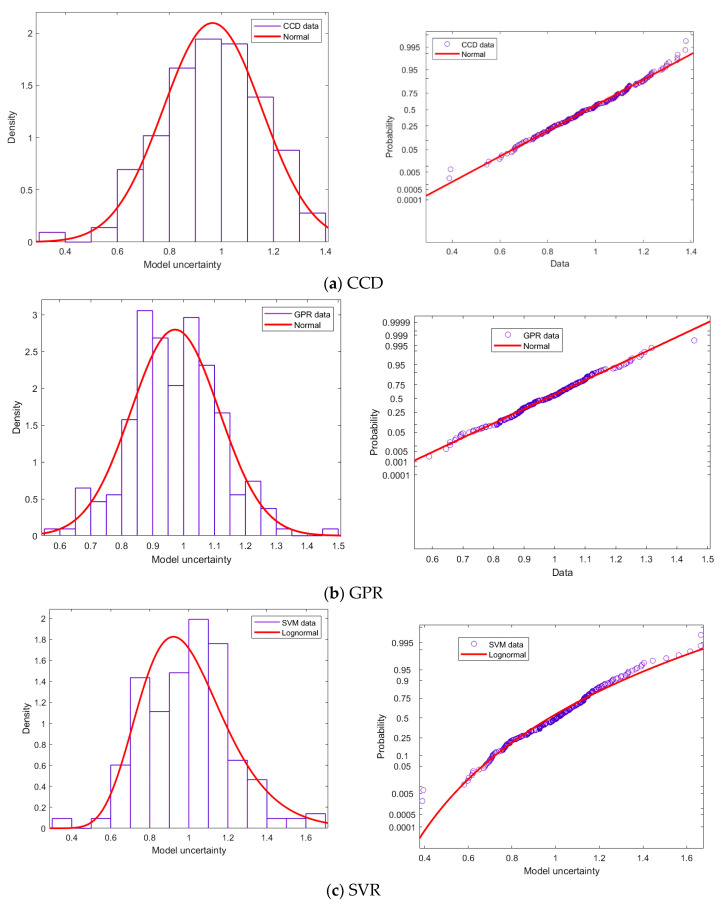
Model uncertainty distribution plots, experimental frequency histogram and probability plots. (**a**) CCD; (**b**) GPR; (**c**) SVR.

A statistical distribution is perceived to be highly skewed if the absolute value of skewness is higher than or close to one [53]. Accordingly, a coefficient of skewness $\eta_{M}$ close to zero suggests that a symmetric, normal distribution is a more appropriate probabilistic description, while lower or higher values indicate that more appropriate distribution functions are negatively or positive skewed (e.g., Lognormal or Weibull type distributions). Hence, the $\eta_{M}$ for the GPR and CCD models, as shown in Table 4, is nearer to what is required from a normal distribution or a negatively skewed distribution. However, the $\eta_{M}$ for the SVR model is nearer to what is required from a basic lognormal distribution. The form of distribution plays a vital role in reliability investigation. The choice of normal and lognormal distribution as the candidate distribution for the model uncertainty was assessed using the Kolmogorov–Smirnov goodness of fit test. It was found that the GPR and CCD models have an underlying normal distribution and the SVR model has a Lognormal distribution, with significance p-values above 0.05. The probability plots and histograms, for all the models, are illustrated in Figure 9. The plots also confirm that the underlying distribution of the GPR and CCD model is the normal distribution, whereas that of the SVR model is the lognormal distribution.

The predictive model, together with its corresponding model uncertainty characteristics demonstrated herein, can be used as the basis for the General Probabilistic Model (GPM) [51] for the tensile breakout resistance in the performance-based design of anchorages. This is specifically possible and it can provide reliable estimates, since the database is comprehensive and it covers a large part of the practical design space. The objective of the GPM is then to provide the unbiased/mean estimate of the resistance, including the expected variability of observations around the mean value. Besides the accuracy in predictions, the GPM must maintain statistical independence between its model uncertainty and the input parameters used to calculate the concrete breakout capacity.

### 5.2. Trends in the Model Uncertainty θ with Basic Input Variables $X_{i}$

**Figure 10:**
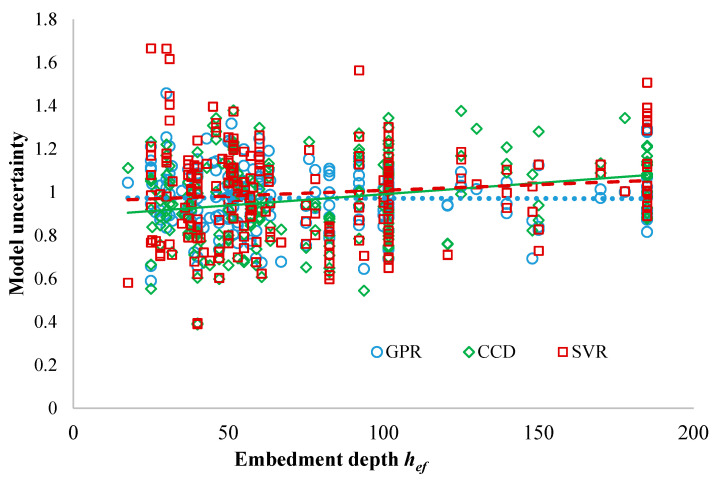

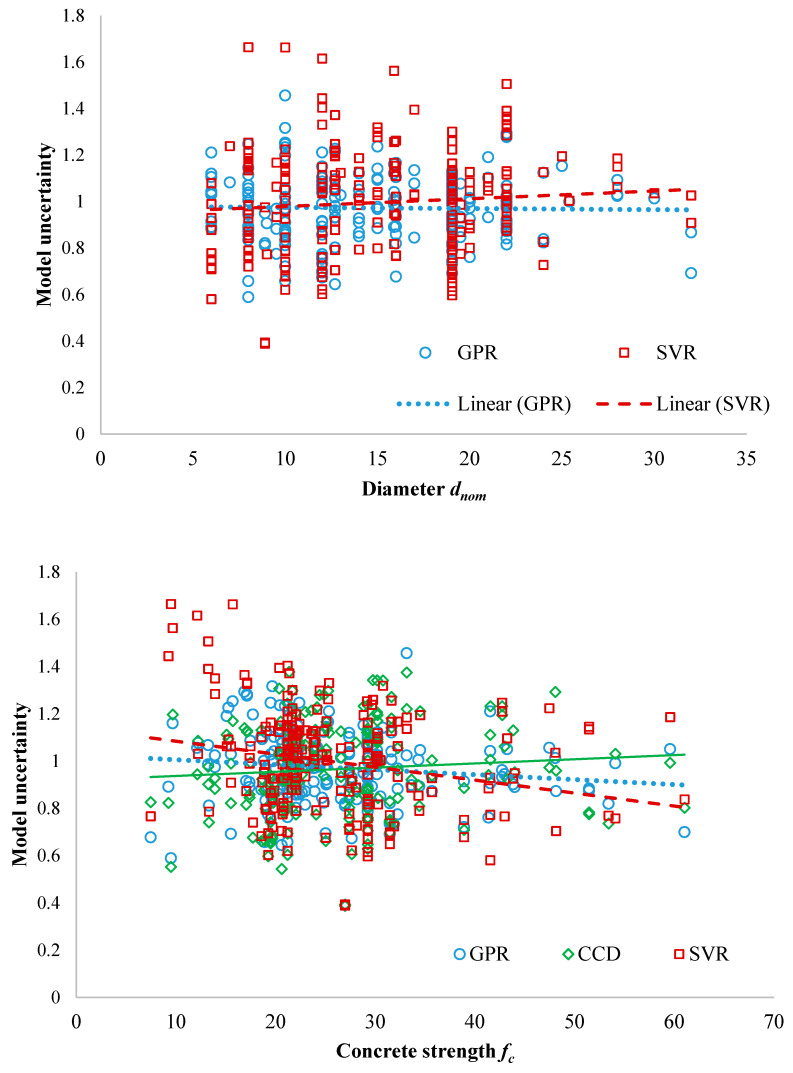
Scatter plots of Model uncertainty versus breakout parameters for all the models.

The influence of basic input variables on model uncertainty θ is assessed by correlating the obtained model uncertainty mean value with input variables (concrete strength, embedment depth and anchor diameter) using the Pearson’s correlation coefficient (r) (Table 5). The Pearson’s correlation coefficient (r) measures the correlation between θ and $X_{i}$. The variation/trend of the model uncertainty variables θ with the basic input variables $X_{i}$ are presented in Figure 10 for the testing dataset. The trends allow assessing whether the proposed models can systematically underpredict or overpredict the actual (experimental) resistance in a way that is correlated with its input parameters [52]. Thus, the most appropriate model to be used as a GPM needs to adequately account for the influences of the input parameters, and maintain small sensitivities with those, i.e., exhibit as weak as possible trends in the parameter’s domain.

As seen from the figures, the GPR and SVR models have no significant trends with the concrete strength, embedment depth and anchor diameter for the range investigated in this study (also see Table 5). On the other hand, the CCD method has some trends with the embedment depth with the correlation value of r=0.25. The anchor diameter is not included as an input variable in the CCD formulation; hence, the CCD model uncertainty variable is not assessed against the anchor diameter $d_{nom}$.

## 6. Conclusions

This contribution proposes ML-based models, which reasonably predict the tensile concrete cone breakout capacity of single anchors given only the attributes. A total of 864 experimental test cases were considered for model development. A total of 75% of the total dataset was used for training and the remaining 25% for testing. Three input variables were considered for model development, and the single output variable was taken as the experimentally observed concrete cone breakout strength. The accuracy of the proposed model was assessed in terms of the mean and coefficient of variation of the ratio of the predicted to experimental breakout strength, *R*^2^, MAE and RMSE. Several machine learning algorithms were trialled in the study, and the GPR and SVR models achieved the best results in terms of highest determination coefficient and the lowest mean square error for both the training and testing dataset. Hence, the GPR and SVR algorithms were adopted for predicting the tensile breakout resistance. The predictions from the developed ML-based models are compared with the predictive accuracy of the CCD method and other soft computing algorithms from previous researchers and developed as part of this investigation.

The assessment conducted in this study is limited to the database of 864 experiments of anchors subjected to tensile loading. While there are few datasets for large diameter anchors, more experimental investigations are recommended to be conducted on large anchors in tension. Based on the investigation, the following conclusions can be made from the current study:

- The GPR and SVR predictions are in good agreement with the experimentally observed tensile capacities. The results demonstrated that the ML-based models learnt and predicted the experimental data reasonably well.
- The assessment revealed that the GPR model yielded the best result with an *R*^2^ value of 0.97 (the closest to the value of one) and MAE values as low as 5.56 (nearest value to zero). It can be said that the GPR model reasonably predicted the experimental data better than the other models investigated.
- The model explainability was also described on the basis of the ML models’ correspondence to existing predictive design equations. Moreover, a very good coincidence has been established between the efficiency of the algorithms predicting the experimental data and the mechanical characteristics of the underlying failure phenomenon. This is ascribed to the Model Explainability based on Analogous Rational and Mechanical phenomena (MEARM), which is for the first time defined and used herein on structural engineering problems.
- Both the SVR and GPR predictive models can technically be used as the basis for establishing probabilistic models GPMs for reliability assessment of fastening design formulations. This is justified by the fact that they deliver low-error predictions, and their predictions are—if at all—only mildly correlated to, and as such biased by the input attributes. These attributes or predictors can be used as stochastic variables. Furthermore, the corresponding model uncertainty characteristics have been quantified.
- The model uncertainty related to the GPR model has a mean value of $\mu_{M}=0.97$(closer to the mean value of 1) and the lowest dispersion of all the models investigated with $\sigma_{M}=0.14$. The model has no major trends with its input parameters; and thus, the most suitable as a GPM for reliability assessment of fastening design formulations. The SVR model has the highest dispersion $\sigma_{M}=0.22$ and the CCD method has some trend with the embedment depth.
- The goodness of fit test indicates that the GPR and CCD model has an underlying normal distribution, whereas the CCD model has a lognormal distribution.

Machine learning-based methods such as the GPR model developed in this study can partially replace finite element modelling/numerical modelling and be implemented as a GPM for reliability analysis. Design concrete breakout resistance obtained from fastening design formulations provided in design standards (such as ACI 318 and EN 1992-4) may then be assessed against the GPM with a view of obtaining the probability of failure or the reliability [51]. This contribution has successfully established that GPR and SVR algorithms can be implemented for an efficient and accurate prediction of the concrete breakout resistance of single anchors in tension and can be an alternative approach for the prediction and estimation of tensile breakout capacity.

## Figures and Tables

**Table 1:** Statistical summary of the experimental database.

Parameters	Symbol	Range				
		Minimum	First Quartile (P25)	Median (P50)	Third Quartile (P75)	Maximum
Concrete cylinder compressive strength (MPa)	$f_{c}$	7.5	20.6	24.1	30.8	63.9
Embedment depth (mm)	$h_{ef}$	17.6	46	66.5	90.3	185
Diameter of anchor (mm)	$d_{nom}$	6	10	15.9	19.5	32
Tensile capacity of anchors (kN)	N	3.3	22.2	40.9	71.8	273.8

**Table 2:** Statistical summary of the training and testing dataset used for the model development.

Statistical Parameter	Datasets	$d_{nom}$ (mm)	$h_{ef}\;(\mathrm{mm})$	$f_{c}\;(\mathrm{MPa})$	N
Range	Training	6 to 32	17.6 to 185	7.5 to 63.9	5.6 to 273.8
	Testing	6 to 32	17.6 to 185	7.5 to 61.0	3.3 to 271.0
Mean	Training	15.7	73.6	26.5	54.5
	Testing	15.2	76.4	26.1	58.3
St. deviation	Training	5.8	32.8	9.6	43.8
	Testing	5.6	45.3	7.5	57.1

**Table 3:** (**a**) Results for various algorithms; (**b**) influence of input variables on Gaussian Process Regression (GPR) and Support Vector Regression (SVR) model performance.

(**a**)							
**Model**		**Training Dataset**			**Testing Dataset**		
		**RMSE (kN)**	***R*^2^**	**MAE (kN)**	**RMSE (kN)**	***R*^2^**	**MAE (kN)**
GPR		8.15	0.97	5.56	10.26	0.97	6.18
SVR		11.73	0.93	7.98	13.12	0.95	8.61
Random Forest (Boosted Trees)		12.1	0.92	7.55	13.33	0.94	8.9
Decision Tree (Fine)		13.4	0.91	7.7	15.5	0.94	9.4
Neural Network		19.2	0.91	14.5	12.5	0.97	8.6
(**b**)							
**Model**	**Input**	**Training Dataset**			**Testing Dataset**		
		**RMSE (kN)**	***R*^2^**	**MAE (kN)**	**RMSE (kN)**	***R*^2^**	**MAE (kN)**
GPR	$d_{nom}$, $f_{c}$, $h_{ef}$	8.15	0.97	5.56	10.26	0.97	6.18
	$f_{c}$, $h_{ef}$	10.7	0.94	6.3	11.7	0.96	7.04
	$d_{nom}$, $h_{ef}$	16.8	0.86	10.9	19.3	0.89	11.2
	$d_{nom}$, $f_{c}$	24.3	0.69	14.3	31.6	0.7	18.4
SVR	$d_{nom}$, $f_{c}$, $h_{ef}$	11.73	0.93	7.98	13.12	0.95	8.61
	$f_{c}$, $h_{ef}$	12.5	0.92	8.2	11.3	0.96	7.9
	$d_{nom}$, $h_{ef}$	17.5	0.84	11.6	19.9	0.88	12.3
	$d_{nom}$, $f_{c}$	31.9	0.47	18.5	45.1	0.53	25.7

**Table 4:** Statistical properties of the resistance models (using the testing dataset).

No.	Parameters	GPR	SVR	CCD
1	*R* ^2^	0.97	0.95	0.96
2	RMSE	10.26	13.12	13.3
3	MAE	6.18	8.61	8.79
4	Mean $\mu_{M}$	0.97	0.99	0.97
5	Standard deviation $\sigma_{M}$	0.14	0.22	0.19
6	Skewness $\left(\eta_{M}\right)$	0.08	0.23	−0.21
7	Minimum ($M_{min}$)	0.59	0.39	0.39
8	Maximum $\left(M_{max}\right)$	1.46	1.38	1.67
9	Ranking for $\mu_{M}$	2	1	2
10	Ranking for $\sigma_{M}$	1	3	2
11	Ranking for $(M_{max}$/$M_{min})$	1	2	3
12	Overall ranking	1.3	2.0	2.3

**Table 5:** Trend analysis of the model uncertainty.

Parameters	GPR	SVR	CCD
Concrete compressive strength $f_{c}$ (MPa)	−0.13	−0.22	0.08
Embedment depth $h_{ef}$ (mm)	−0.001	0.11	0.25
Diameter of anchor $d_{nom}$ (mm)	−0.01	0.08	-

## Data Availability

The data presented in this study are available on request from the corresponding author.

## Nomenclature

$\sigma_{M}$	standard deviation of the prediction error
$\mu_{M}$	mean value of the prediction error
$c_{1}$	edge distance of a single anchor
$d_{nom}$	anchor diameter
r	Pearson’s correlation coefficient
$f_{c}$	is the concrete compressive strength.
$k_{ucr}$	is a product-specific value for non-cracked concrete
$f_{ck,cube}$	is the characteristic compressive strength from 150 mm concrete cube specimens
$f_{cm}$	mean value of concrete compressive cylinder strength
$f_{cc}$	is the mean compressive strength measured at 200 mm concrete cube specimens
$l_{f}$	is the influence length of the anchor loaded in shear
$k_{c}$	is equal to the value of 13.5 for metal expansion anchors and bonded anchors
$\alpha_{c}$	is equal to the value of 1.0 for an undisturbed uniaxial compression stress state (this occurs for $h_{ef}$ equal to approximately 5 anchor diameters) is equal to the value of 0.8 for anchors within compression zones with cracks developing parallel to the compression direction (this occurs for $h_{ef}$ greater than 5 anchor diameters)
$k_{1},\;k_{2},\;k_{3}$	are calibration factors
$h_{ef}$	is the anchor embedment depth (the distance of the failure invitation point to the free surface)
$V_{Rm,c}^{0}$	mean shear breakout resistance
N	tensile breakout resistance
$N_{Rm,c}^{0}$	mean tensile breakout resistance
$N_{Rk,c}^{0}$	characteristic tensile breakout resistance
$N_{exp,x}$	experimentally observed failure load for a single anchor test x in tension
X	represents the input variable set which include the embedment depth $h_{ef}$, anchor $d_{nom}$ and the concrete strength $f_{c}$
$N_{pred,\;x}$	represents mean concrete breakout model prediction for the same anchor test x
